# Post-stroke dysphagia and ambient air pollution are associated with dementia

**DOI:** 10.3389/fnagi.2023.1272213

**Published:** 2023-10-10

**Authors:** Kuo-Wei Lee, Hao-Wei Chung, Hui-Min Hsieh, Yu-Hsiang Tsao, Chih-Hsien Hung, Ming-Chu Feng, Chih-Hsing Hung

**Affiliations:** ^1^Graduate Institute of Medicine, College of Medicine, Kaohsiung Medical University, Kaohsiung, Taiwan; ^2^Department of Neurology, Kaohsiung Municipal Siaogang Hospital, Kaohsiung Medical University, Kaohsiung, Taiwan; ^3^Department of Neurology, Kaohsiung Medical University Hospital, Kaohsiung Medical University, Kaohsiung, Taiwan; ^4^Department of Pediatrics, Kaohsiung Medical University Chung Ho Memorial Hospital, Kaohsiung Medical University, Kaohsiung, Taiwan; ^5^Department of Biological Science and Technology, National Yang Ming Chiao-Tung University, Hsinchu, Taiwan; ^6^Department of Pediatrics, Kaohsiung Municipal Siaogang Hospital, Kaohsiung, Kaohsiung Medical University, Kaohsiung, Taiwan; ^7^Department of Public Health, College of Health Sciences, Kaohsiung Medical University, Kaohsiung, Taiwan; ^8^Division of Medical Statistics and Bioinformatics, Department of Medical Research, Kaohsiung Medical University Hospital, Kaohsiung, Taiwan; ^9^Center for Big Data Research, Kaohsiung Medical University, Kaohsiung, Taiwan; ^10^School of Medicine, College of Medicine, Kaohsiung Medical University, Kaohsiung, Taiwan; ^11^Department of Dysphagia Functional Reconstructive Center, Kaohsiung Municipal Siaogang Hospital, Kaohsiung Medical University, Kaohsiung, Taiwan; ^12^Department of Nursing, Fooyin University, Kaohsiung, Taiwan; ^13^Research Center for Environmental Medicine, Kaohsiung Medical University, Kaohsiung, Taiwan; ^14^Department of Pediatrics, Faculty of Pediatrics, College of Medicine, Kaohsiung Medical University, Kaohsiung, Taiwan

**Keywords:** stroke, dysphagia, dementia, air pollution, elderly

## Abstract

**Introduction:**

This cohort study aimed to explore the potential association between ambient air pollution and dementia incidence in adults who have experienced a stroke. Additionally, the study aimed to determine dysphagia as a predictive factor for the subsequent development of dementia in patients with stroke.

**Materials and methods:**

This retrospective nested case–control study used data from the Kaohsiung Medical University Hospital Database in Taiwan. Data collected include average ambient air pollution concentrations within 3 months and 1 year after the index dysphagia date. The primary outcome includes incident dementia in patients with or without dysphagia. Logistic regression analysis was performed to examine the association between significant air pollution exposure and the risk of dementia while controlling for baseline demographic characteristics (age and sex), and comorbidities.

**Results:**

The univariable regression models revealed a higher likelihood of dementia diagnosis in patients with dysphagia (odds ratio = 1.493, 95% confidence interval = 1.000–2.228). The raw odds ratios indicated a potential link between air pollution exposure and elevated dementia risks in the overall study population and patients with stroke without dysphagia, except for O_3_. Particulate matter (PM)2.5 and nitrogen oxides (NOx) exhibited significant effects on the risk of dementia in the stepwise logistic regression models.

**Conclusion:**

The presence of dysphagia following a stroke may pose a risk of developing dementia. Additionally, PM2.5 and NOx exposure appears to elevate the risk of dementia in patients with stroke.

## 1. Introduction

Stroke is the leading cause of disability and mortality and is a major burden globally (GBD 2019 Stroke Collaborators, 2021). The risk of dementia, which is another disabling disorder, increases after a stroke. A previous meta-analysis conducted in 2009 concluded a 7.4 and 12% prevalence of dementia in population-based and hospital-based studies of first-ever stroke, respectively, in which pre-stroke dementia was excluded (Pendlebury and Rothwell, 2009). Another subsequent meta-analysis that include12 studies conducted in 2018 established the pooled risk ratio for all-cause dementia of 2.18 for the stroke incidence (Kuźma et al., 2018). Additionally, several characteristics and complications of stroke have been reported as strong predictors for post-stroke dementia, including stroke severity, infarct volume, number of strokes separated in space and time, dysphasia, incontinence, and early seizure (Pendlebury and Rothwell, 2009; Koton et al., 2022).

Dysphagia is a frequently occurring clinical disease in the elderly, and it causes malnutrition, aspiration pneumonia, and decreased quality of life. Patients with neurodegenerative diseases, such as Parkinson’s disease and dementia, may develop dysphagia (Takizawa et al., 2016). Conversely, patients with dysphagia may demonstrate an increased risk of developing dementia. A cross-sectional study recruited 415 people aged ≥65 years in Korea and assessed their swallowing and cognitive functions. Participants with dysphagia were significantly more likely to have non-amnestic mild cognitive impairment than those without dysphagia (Yang et al., 2014). The occurrence of dysphagia after a stroke could elevate the likelihood of developing dementia because dysphagia is a commonly encountered complication following a stroke. A cross-sectional study included 55 patients who experienced their first stroke and retrospectively categorized them into two groups based on the presence or absence of dysphagia. The study revealed that the dysphagia group exhibited inferior cognitive performance (Jo et al., 2017). However, a large cohort study to evaluate the association between post-stroke dysphagia and dementia remains currently lacking.

Recently ambient air pollution has received considerable attention. Particulate air pollution, including particulate matter (PM)10 and PM2.5, and gaseous pollutants, including nitrogen oxides (NOx), sulfur dioxide (SO_2_), carbon monoxide (CO), and ozone (O_3_), were reported to be associated with dementia (Tsai et al., 2019; Hahad et al., 2020; Wood et al., 2022). A recent meta-analysis of 17 studies reported a 3% increase in the risk of dementia per 1 μg/m^3^ increment in PM2.5. The connection between dementia and NOx and O_3_ levels was less definite, although a substantial link cannot be dismissed, and a considerable heterogenicity was found in the study results (Abolhasani et al., 2023). Thus, the association between gaseous pollutants and dementia remains unestablished.

This retrospective cohort study aimed to examine the potential association between ambient air pollution and the onset of dementia in adults who have had a stroke. Additionally, the study aimed to determine whether dysphagia as a predictive factor for subsequent dementia development in patients with stroke.

## 2. Materials and methods

### 2.1. Study design and data source

This retrospective nested case–control study examined the association between air pollution exposure and risks of dementia in patients with stroke with and without dysphagia. This study used the Kaohsiung Medical University Hospital Database (KMUHRD), which consists of patient electronic medical records from Kaohsiung Medical University (KMU) health system affiliated hospitals (one medical center, two regional hospitals, and one local community hospital) in southern Taiwan. The Division of Medical Statistics and Bioinformatics, Department of Medical Research, Kaohsiung Medical University Hospital managed the database. All personal identifiers are removed from data in the KMUHRD following the Personal Information Protection Act in Taiwan. The International Classification of Diseases, ninth revision, Clinical Modification (ICD-9-CM) is used to record diagnoses in medical records before 2016 and the ICD, tenth revision (ICD-10) is used in the medical records after 2016. The ICD code within KMUHRD is interconnected with Taiwan’s national health insurance system. All insurance claims must undergo meticulous scrutiny by medical reimbursement specialists and peer review based on established diagnostic criteria. In cases where healthcare professionals or hospitals make incorrect diagnoses or coding errors, they may face substantial penalties. Consequently, the utilization of ICD-9 and ICD-10 codes for validating diagnoses within KMUHRD is highly dependable. Air Quality Management Center, Environmental Protection Bureau, Kaohsiung City Government collected the air quality monitoring data. At present, the Environmental Protection Agency has 8, 2, 1, and 1 general, traffic, industrial, and background air quality monitoring stations, respectively, in Kaohsiung City. The monitoring items include carbon monoxide, sulfur dioxide, ozone, two NOx, suspended particulates, etc., that are continuously monitored for 24 h. The Institutional Review Board of the KMUH (IRB number: KMUHIRB-E(I)-20200002) approved this study and waived the written informed consent requirement because of the retrospective design and use of deidentified data.

### 2.2. Study population

**Figure 1:**
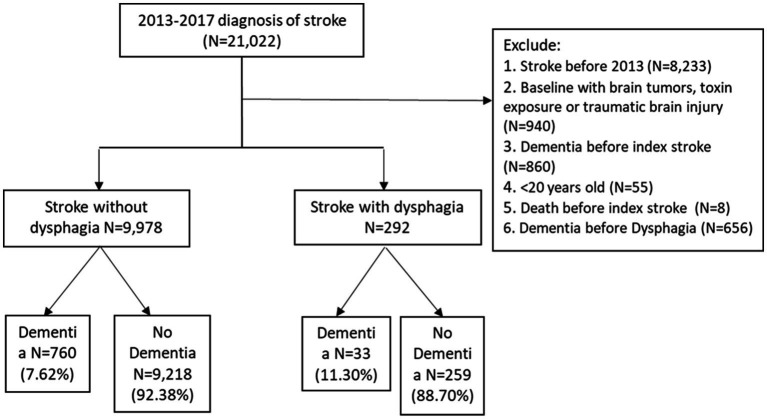
Study flowchart of the inclusion and exclusion criteria.

The inclusion criteria for the study were as follows: (1) Patients with newly diagnosed stroke (ICD-9-CM code: 430–432, 433.x1, 434.x1, 436 or ICD-10-CM code: I60–I65, I67.89, I67.9) having at least three outpatient visits or at least one inpatient admission in the KMUHRD records during the patient identification period from 2013 to 2017; (2) the naïve first date of stroke was defined as the index stroke date; and (3) we classified patients with stroke into two groups. The group with dysphagia (ICD-9-CM code:438.82, 432.46, 437.50, 507.0, 787.2, or ICD-10-CM code: R13.0, R13.10–R13.19, I69.091, I69.191, I69.291, I69.391, I69.891, I69.991, and J69.0) and the group without any medical history of dysphagia. We randomly assigned a proxy date of index dysphagia for patients without dysphagia. The exclusion criteria included any stroke diagnosed before 2013, with brain tumors, toxin exposure, or traumatic brain injury, having any dementia diagnosed before index stroke or index dysphagia date, patients younger than 20 years old, and those who died before the index stroke. Finally, This study included 10,270 patients, including 292 and 9,978 patients with and without dysphagia, and tracked the occurrence of incident dementia among those patients until 2018. Figure 1 shows the study flowchart of the inclusion and exclusion criteria.

### 2.3. Measurements

The primary outcome of interest was the incident dementia based on the diagnosis codes (ICD-9-CM code: 290.x, 291.2, 292.82, 294.1x, 294.2x, 331.0, 331.19, and 331.82, or ICD-10-CM code: F01–F05, G30, G30.x, and G31). Variables included age, sex, and comorbidities. Age was stratified into four groups, including <55 years, 55–64 years, 65–74 years, and ≧75 years. Comorbidities are identified by ICD-9-CM and ICD-10 coding methods, which are recorded if they occurred in the year before the index date. Comorbidities and their corresponding codes (ICD-9-CM; ICD-10-CM codes) included hypertension (401–405; I10–I13, and I15), hyperlipidemia (272; E75, E77, E78, and E88), diabetes mellitus (250; E08.x, E09.x, E10.x, E11.x, and E13.x), myocardial infarction (410.x and 412.x; I21.x, and I25.x), atrial fibrillation (427.31; I48), depression (296.2, 296.3, and 311; F32), and epilepsy (345; G40). This study used the Deyo-Charlson Comorbidity Index (CCI), which is an ICD-9-CM coding adaption to identify levels of overall chronic comorbidity.

For air pollution exposure measurement, we conducted an analysis of daily (24-h) average concentrations of various air quality parameters, including PM2.5, PM10, SO_2_, O_3_, NO, NO_2_, and NOx levels, which were gathered from air quality monitoring stations located throughout Kaohsiung city. The address of each patient at the district level was utilized to confirm the proximity of the nearest air quality monitoring station. We then calculated the average cumulative air pollution exposure over 3 months and 1 year following the index dysphagia date for all study participants. To ensure comparability of cumulative air pollution exposure levels across different time periods for each patient, we employed the cumulative air pollution per interquartile range (IQR) as an adjustment factor to account for mean variability among multiple datasets.

### 2.4. Statistical analysis

The chi-square test was used to compare the distribution of sociodemographic characteristics and comorbidity between the two groups. The association between major air pollution exposure and the risk of dementia was analyzed by logistic regression while controlling for baseline demographic characteristics (age and sex) and aforementioned comorbidities. Odds ratios (OR) and 95% confidence intervals (CIs) were calculated to assess the risk of dementia development in different cumulative air pollution exposure periods. The SAS statistical software version 9.4 (SAS Institute, Cary, NC, USA) was used for all statistical analyses. All statistical tests were 2-sided, and a 2-tailed *p*-value of <0.05 was considered significant.

## 3. Results

Table 1 shows the descriptive results of patient demographic and clinical statistics between patients with stroke with and without dysphagia. Patients with newly diagnosed dysphagia had a mean age of 67.39 (±13.18) years, 59.59% were male, 39.73% had hypertension, and 52.74% have CCI scores of >5. Table 2 presents the descriptive results of air pollution exposure distribution within 3 months and 1 year after the index dysphagia date among the study subjects. The mean value and standard deviation of 1-year air pollutants for PM10, PM2.5, NO, NO_2_, CO, SO_2_, NOx, and O_3_ were 65.17 (±9.54), 30.60 (±5.97), 4.28 (±1.37), 19.02 (±3.20), 0.51 (±0.09), 5.26 (±1.46), 23.31 (±4.44), and 27.58 (±2.94), respectively.

**Table 1:** Descriptive results of patient demographic and clinical statistics between patients with stroke with and without dysphagia.

	Dysphagia	Non-dysphagia	
*N*	292	9,978	*p*-value
Age for stroke (Mean ± SD)	67.39 (±13.18)	66.67 (±13.64)	0.373
<55	48 (16.44%)	1,876 (18.80%)	0.393
55–64	82 (28.08%)	2,417 (24.22%)	
65–74	72 (24.66%)	2,652 (26.58%)	
> = 75	90 (30.82%)	3,033 (30.40%)	
Gender (*N*, %)			0.465
Female	118 (40.41%)	4,246 (42.55%)	
Male	174 (59.59%)	5,732 (57.45%)	
Comorbidities (*N*, %)			
Hypertension	116 (39.73%)	3,927 (39.36%)	0.899
Diabetes	61 (20.89%)	2,141 (21.46%)	0.816
Hyperlipidemia	26 (8.90%)	1,537 (15.40%)	0.002
Myocardial infarction	3 (1.03%)	175 (1.75%)	0.349
Depression	6 (2.05%)	168 (1.68%)	0.628
Epilepsy	16 (5.48%)	226 (2.26%)	<0.001
Charlson comorbidity score (Mean ± SD)	5.10 (±2.92)	4.23 (±2.98)	<0.001
0–2	47 (16.10%)	3,387 (33.94%)	<0.001
3–4	91 (31.16%)	2,894 (29.00%)	
5+	154 (52.74%)	3,697 (37.05%)	

**Table 2:** Descriptive results of distribution of air pollution exposure within 3 months and 1 year post to index dysphagia date among study subjects.

Pollutants	Value	Min	Percentile			Max	IQR
			25%	50%	75%		
Within 3 months after Dysphagia (mean ± sd)							
PM10	63.36 ± 23.27	21.33	60.00	83.00	117.00	43.33	39.67
PM2.5	29.28 ± 13.15	5.00	28.33	39.67	65.33	17.33	22.33
NO	4.16 ± 1.89	0.87	3.76	4.94	15.82	2.89	2.05
NO_2_	18.57 ± 6.25	3.19	17.82	23.30	35.21	13.63	9.67
CO	0.50 ± 0.15	0.12	0.48	0.61	1.05	0.37	0.23
SO_2_	5.12 ± 1.67	1.20	4.67	6.13	10.80	3.97	2.17
NOx	22.72 ± 7.77	4.27	21.22	27.81	49.13	16.72	11.09
O_3_	27.48 ± 5.67	5.67	27.10	31.60	45.73	23.63	7.97
Within 1 year after Dysphagia (mean ± sd)							
PM10	65.17 ± 9.54	37.08	66.75	71.67	87.38	58.00	13.67
PM2.5	30.60 ± 5.97	17.58	27.92	34.25	49.31	26.67	7.58
NO	4.28 ± 1.37	1.16	4.03	5.06	9.13	3.31	1.75
NO_2_	19.02 ± 3.20	6.46	18.80	21.45	26.48	16.80	4.64
CO	0.51 ± 0.09	0.29	0.50	0.56	0.82	0.45	0.11
SO_2_	5.26 ± 1.46	1.66	4.88	6.17	10.13	4.34	1.83
NOx	23.31 ± 4.44	7.71	22.92	26.35	35.61	20.47	5.88
O_3_	27.58 ± 2.94	15.40	27.12	29.59	35.97	25.96	3.63

Min, minimum; max, maximum; IQR, interquartile range.

Table 3 shows the crude OR of the association between air pollution per interquartile range (IQR) and risk of dementia by exposure periods following the index dysphagia date among all study subjects, and with/without dysphagia. The crude ORs indicated that air pollution exposure may be associated with greater risks of dementia for overall study subjects and patients with stroke without dysphagia, except for O_3_, while the effects were all insignificant among patients with stroke with dysphagia. Table 4 further presents the results from univariable and multivariable logistic regressions for examining the factors associated with the risk of dementia, while controlling for different factors. The univariable regression models demonstrated that patients with dysphagia were more likely diagnosed with dementia (OR = 1.493, 95% CI = 1.000, 2.228). After controlling for confounding factors (as model 1 to model 5), dysphagia became insignificant associated with dementia (adjusted OR = 1.353, 95% CI = 0.894, 2.047). Additionally, patients with older age, epilepsy disease, and higher CCI scores had a greater risk of dementia in both univariable and multivariable results. Regarding the types of air pollution exposure, the results from stepwise logistic regression models in model 5 (Table 4) revealed that PM2.5 (aOR = 1.053, 95% CI = 1.04, 1.067) and NOx (aOR = 1.041, 95% CI = 1.021, 1.061) had greater effects on the risk of dementia.

**Table 3:** Crude odds ratio of the association between air pollution per IQR and risk of dementia by exposure periods post to index dysphagia date.

	Dysphagia group			Non-dysphagia group			All study subjects		
	Crude OR	95%CI	*p*-value	Crude OR	95%CI	*p*-value	Crude OR	95%CI	*p*-value
Air pollution index (per IQR)									
Three-month after Dysphagia									
PM10	1.213	0.636–2.314	0.558	1.287	1.124–1.474	<0.001	1.283	1.124–1.465	<0.001
PM2.5	1.455	0.745–2.843	0.272	1.386	1.212–1.586	<0.001	1.387	1.216–1.582	<0.001
NO	1.039	0.636–1.697	0.878	1.215	1.126–1.310	<0.001	1.208	1.121–1.302	<0.001
NO_2_	1.211	0.654–2.241	0.543	1.299	1.150–1.468	<0.001	1.294	1.148–1.458	<0.001
CO	1.156	0.627–2.132	0.642	1.29	1.147–1.450	<0.001	1.282	1.143–1.438	<0.001
SO_2_	1.363	0.837–2.218	0.213	1.346	1.222–1.484	<0.001	1.347	1.225–1.482	<0.001
NOx	1.027	0.564–1.870	0.929	1.281	1.147–1.429	<0.001	1.269	1.139–1.414	<0.001
O_3_	1.327	0.760–2.317	0.32	1.052	0.940–1.177	0.38	1.062	0.951–1.185	0.288
One-year after Dysphagia									
PM10	0.956	0.551–1.661	0.874	1.672	1.467–1.905	<0.001	1.629	1.435–1.851	<0.001
PM2.5	1.403	0.893–2.205	0.142	1.736	1.581–1.907	<0.001	1.721	1.570–1.887	<0.001
NO	1.251	0.776–2.019	0.358	1.391	1.267–1.526	<0.001	1.384	1.263–1.517	<0.001
NO_2_	1.180	0.639–2.179	0.597	1.555	1.371–1.763	<0.001	1.539	1.361–1.740	<0.001
CO	0.977	0.586–1.629	0.929	1.336	1.214–1.471	<0.001	1.321	1.202–1.452	<0.001
SO_2_	1.351	0.844–2.164	0.211	1.424	1.298–1.561	<0.001	1.422	1.298–1.556	<0.001
NOx	1.202	0.700–2.063	0.505	1.471	1.321–1.638	<0.001	1.460	1.314–1.622	<0.001
O_3_	1.097	0.636–1.890	0.740	1.011	0.909–1.123	0.842	1.014	0.915–1.125	0.786

**Table 4:** Results from univariable and multivariable logistic regression models for the risk of incident dementia among stroke patients.

	Univariable	Model 1	Model 2	Model 3	Model 4	Model 5
	OR (95%CI)	aOR (95%CI)	OR (95%CI)	OR (95%CI)	OR (95%CI)	OR (95%CI)
Dysphagia (ref: no)						
yes	1.493 (1.000,2.228)^*^	1.459 (0.971,2.193)	1.364 (0.906,2.055)	1.353 (0.894,2.047)	1.341 (0.886,2.03)	1.332 (0.88,2.015)
Age (ref:<55)						
55–64	2.395 (1.607,3.568)^***^	2.375 (1.594,3.539)^***^	2.304 (1.544,3.438)^***^	2.325 (1.554,3.476)^***^	2.312 (1.547,3.457)^***^	2.299 (1.538,3.436)^***^
65-74	4.164 (2.858,6.065)^***^	4.097 (2.812,5.969)^***^	3.876 (2.654,5.662)^***^	3.973 (2.715,5.814)^***^	3.925 (2.684,5.738)^***^	3.914 (2.677,5.722)^***^
> = 75	7.46 (5.193,10.716)^***^	7.226 (5.027,10.388)^***^	6.799 (4.713,9.809)^***^	6.833 (4.728,9.876)^***^	6.731 (4.662,9.719)^***^	6.724 (4.658,9.706)^***^
Gender (ref: female)						
male	0.714 (0.610,0.836)^***^	0.804 (0.685,0.944)^**^	0.782 (0.666,0.919)^**^	0.798 (0.678,0.939)^**^	0.798 (0.679,0.939)^**^	0.798 (0.678,0.938)^**^
Hypertension (ref: no)						
yes	1.062 (0.905,1.245)	-	0.871 (0.739,1.027)	0.866 (0.733,1.024)	-	
Epilepsy (ref: no)						
yes	1.749 (1.160,2.638)^**^	-	1.903 (1.244,2.913)^**^	1.903 (1.237,2.928)^**^	1.892 (1.231,2.908)^**^	1.878 (1.222, 2.888)^**^
CCI score						
	1.09 (1.065,1.116)^***^	-	1.056 (1.032,1.081)^***^	1.063 (1.036,1.09)^***^	1.06 (1.033,1.087)^***^	1.060 (1.033,1.087)^***^
Air pollution						
PM10	1.031 (1.022,1.04)^***^	-	-	1.009 (0.995,1.024)	-	-
PM2.5	1.066 (1.053,1.078)^***^	-	-	1.037 (1.015,1.059)^***^	1.054 (1.04,1.067)^***^	1.053 (1.04,1.067)^***^
NO	1.221 (1.157,1.288)^***^	-	-	1.286 (0.918,1.803)	1.135 (1.071,1.202)^***^	-
NO2	1.096 (1.067,1.125)^***^	-	-	1.016 (0.732,1.412)	-	-
CO	15.627 (6.603,36.983)^***^	-	-	5.31 (0.795,35.453)	-	-
SO2	1.213 (1.153,1.276)^***^	-	-	1.111 (0.937,1.317)	-	-
NOx	1.068 (1.049,1.088)^***^	-	-	0.910 (0.67,1.238)	-	1.041 (1.021,1.061)^***^
O3	0.988 (0.962,1.014)	-	-	1.008 (0.961,1.057)	-	-

The air pollution values in the regression model were recorded within 1 year after dysphagia. Model 1 was a multivariable logistic regression model for the association between dysphagia and risk of dementia while controlling for age and gender categories; model 2 was controlled for age, gender and comorbidities; model 3 was controlled for age, gender, comorbidities, and air pollutants; model 4 was stepwise regression model; model5 was controlled for age, gender, comorbidities, PM2.5 and NOx air pollutions.

*** *p* < 0.001.

** *p* < 0.01.

* *p* < 0.05.

## 4. Discussion

To our knowledge, this is the first cohort study investigating the relationship between post-stroke dysphagia and dementia. Patients with dysphagia after a stroke exhibited inferior cognitive performance corresponding to a previous study. Further, our findings indicate that patients with dysphagia after a stroke have a higher risk of developing dementia. Earlier research revealed that suffering from a stroke in the left hemisphere escalates the likelihood of developing dementia (Pendlebury and Rothwell, 2009; El-Sheik et al., 2021). Additionally, a study investigating swallowing lateralization revealed that the left hemisphere is crucial for the volitional aspects of swallowing and the oral phase (Daniels et al., 2006). However, a study examining the correlation between acute stroke lesion locations and the impairment of various aspects of swallow physiology revealed that different post-stroke swallowing functions related to distinct lesion locations, primarily in the right hemisphere (Wilmskoetter et al., 2019). The location of the infarction could contribute to the relationship between post-stroke dysphagia and dementia although the exact mechanism remains unclear.

Though the underlying mechanism of post-stroke dysphagia related to dementia remains unclear, it is apparent that dysphagia can have multifaceted effects. Not only can it result in inadequate food intake leading to malnutrition, but it can also contribute to depression and non-adherence to oral medication, potentially exacerbating the control of underlying diseases. These conditions may increase the risk of developing dementia. Therefore, for individuals with post-stroke dysphagia, nutritional support, such as enteral feeding or dietary modifications, is of paramount importance. Monitoring medication adherence and considering proper medication management, such as using crushed or liquid formulations, is essential. Additionally, providing mental support is crucial to prevent depression.

Our study revealed that the risk of dementia increases with advancing age, which is consistent with previous studies. A meta-analysis of eight studies revealed a higher incidence of dementia in individuals aged >80 years (risk ratio [RR] = 4.66, 95% CI = 2.36–9.22) and those aged 70–79 years (RR = 2.68, 95% CI = 1.52–4.74) compared to those aged 60–69 years (Surawan et al., 2017). Another large prospective cohort study conducted in the United Kingdom, comprising 2,305 patients with stroke or transient ischemic attack, revealed that even after adjusting for age, sex, education, and severity of an event, age of ≥75 years remains a significant predictor of developing dementia (hazard ratio [HR] = 4·58, 95%CI = 3.62–5.80; Pendlebury and Rothwell, 2019). Furthermore, a recent nationwide cohort study in Taiwan, involving 8,236 patients with stroke and 8,236 matching non-stroke patients, reported that individuals aged ≥60 years had a substantial risk of post-stroke dementia (HR = 3.03, 95% CI = 2.39–3.84; Li et al., 2019). We also observed gender differences in dementia development, with females being at a higher risk, which is in line with previous studies. A previous meta-analysis study concluded that females were a predictor of post-stroke dementia (OR = 1.3, 95% CI = 1.1–1.6; Pendlebury and Rothwell, 2009). Another meta-analysis study conducted in 2020, which included 47 studies investigating the prevalence of dementia, reported a higher prevalence of dementia in women than men, with 788 cases versus 561 cases per 10,000 persons in the overall analysis (Cao et al., 2020).

Additionally, we discovered that epilepsy is a robust predictor of post-stroke dementia, which is consistent with previous research. A recent extensive cohort study that analyzed data from The IBM Watson Health MarketScan Commercial Claims and Encounters database identified 23,680 patients with stroke aged 18–60 years. This study revealed that patients with stroke who experienced seizures had a significantly higher risk of developing dementia compared to those without seizures (HR = 2.53, 95% CI = 1.84–3.48; Lekoubou et al., 2022). Another retrospective multicenter cohort study conducted in the United States of America, involving 292,262 veterans, revealed that 2,166 of them developed late-onset unprovoked seizures. The study revealed that veterans with seizures had a higher risk of dementia compared to those without seizures (HR = 1.89, 95% CI = 1.62–2.20; Keret et al., 2020).

The current study revealed significant associations between exposure to PM2.5 and the incidence of dementia in patients with stroke, even after adjusting for age, gender, and various comorbidities. These results are consistent with previous research which has revealed that exposure to particulate air pollution, particularly PM2.5, increases the risk of developing dementia (Smargiassi et al., 2020; Yan et al., 2022; Abolhasani et al., 2023). A large national population-based cohort study conducted in Taiwan revealed that an increase in the PM2.5 concentration by one IQR (10.29) elevated the risk of dementia by approximately 5% (HR = 1.05, 95% CI = 1.04–1.05) (Yan et al., 2022). Another large cohort study in Canada involving 1,807,133 individuals revealed that an IQR increase in time-varying exposure to PM2.5 (IQR = 3.90 μg/m^3^) was associated with an HR of 1.016 (95% CI = 1.003–1.028; Smargiassi et al., 2020). The underlying mechanism of fine particulate air pollution-related dementia remains unclear, but it has been hypothesized that neuroinflammation occurs in response to air pollution, which is related to neurodegeneration (Jayaraj et al., 2017). A previous study has revealed that PM2.5 penetration across the blood–brain barrier initiates astrogliosis, causing slight neuronal loss and microglial infiltration. Furthermore, proinflammatory mediators and NO released from the M1 microglia further exacerbate neuronal damage (Kang et al., 2021). Additionally, our study reveals an increased risk of developing dementia following a stroke in individuals exposed to higher NOx concentrations, which is consistent with a prior research. A longitudinal study conducted in Sweden that examined the association between long-term exposure to traffic-related air pollution and dementia incidence revealed that individuals in the highest exposure group to NOx were more likely to be diagnosed with dementia compared to those in the lowest exposure group (HR = 1.43, 95% CI = 0.998–2.05; Oudin et al., 2016). Furthermore, another large cohort study that utilized data from UK Biobank reported that NOx was associated with a greater risk of any dementia incidence (HR = 1.11, 95% CI = 1.06–1.17 for a 1 IQR increase; Parra et al., 2022).

The current study has several limitations. First, some confounding factors cannot be obtained from our database which might have affected the development of dementia, such as smoking, family history, nutrition status, education status, and location of infarction regions. Nonetheless, our statistical findings did confirm that the sample size was adequate for establishing a meaningful interpretation of the phenomena observed in this study. Second, the use of a database as a source of dementia data may contribute to bias in terms of the under-detection of dementia in those with an undiagnosed condition although the prevalence of post-stroke dementia in the present study is similar to previous reports. Third, the air pollution data in the current study mainly includes outdoor pollutants, and the decreased outdoor activity in people with severe stroke may exclude the evaluation of the effect of indoor pollutants on developing dementia. Fourth, the results cannot be generalized because we only included people from Taiwan, and the prevalence of post-stroke cognitive decline and dementia varies among different ethnicities (Clark et al., 2018). Fifth, while vascular dementia is the predominant subtype of post-stroke dementia (Mijajlović et al., 2017), this study lacks precise information about dementia subtypes. One contributing factor to this gap could be the variability in how doctors in this hospital attribute ICD codes.

## 5. Conclusion

Our findings indicate that post-stroke dysphagia may elevate the risk of dementia; however, this association could be influenced by other confounding factors, including age, gender, and comorbidities. Additionally, PM2.5 and NOx exposure increases the risk of dementia development in patients with stroke. Our study emphasizes the importance of providing increased attention to patients with dysphagia after a stroke to mitigate the risk of future dementia and minimize exposure to air pollutants. Further research is necessary to assess the underlying mechanisms involved.

## Data availability statement

The original contributions presented in the study are included in the article/supplementary material, further inquiries can be directed to the corresponding authors.

## Ethics statement

The studies involving humans were approved by Institutional Review Board of Kaohsiung Medical University Hospital. The studies were conducted in accordance with the local legislation and institutional requirements. The ethics committee/institutional review board waived the requirement of written informed consent for participation from the participants or the participants’ legal guardians/next of kin because the retrospective design and use of deidentified data.

## Author contributions

K-WL: Conceptualization, Data curation, Writing – original draft, Writing – review & editing. H-WC: Conceptualization, Data curation, Writing – original draft. H-MH: Data curation, Formal Analysis, Methodology, Writing – original draft. Y-HT: Writing – original draft. C-HsieH: Writing – review & editing. M-CF: Resources, Supervision, Validation, Writing – review & editing. C-HsinH: Conceptualization, Funding acquisition, Resources, Supervision, Validation, Writing – review & editing.

## Abbreviations

CCI: Charlson comorbidity index
OR: Odds ratios
CI: Confidence interval
PM: Particulate matter
NO: Nitric oxide
NOx: Nitrogen oxides
CO: Carbon monoxide
SO_2_: Sulfur dioxide
HR: Hazard ratios
